# Closed Ward Reopened at Norwich

**Published:** 1921-06-18

**Authors:** 


### Closed Ward Reopened at Norwich.

It is pleasing to note that the finances of the Norfolk and Norwich Hospital have so greatly improved since the end of last year, when the position was decidedly serious, that the ward which was "closed for want of funds" is now to be reopened. The overdraft at the end of December was over £28,000, and the expenditure at that time exceeded the income by £250 each week. Although the overdraft still remains, the income received to date is sufficient to meet the current expenditure, and the Board of Management has decided to open all the wards in the hope that the accumulated deficit will eventually be cleared off. The advisability of closing a ward as a stimulus to obtaining funds for hospitals is open to question, but there is no doubt that the closing of a ward at Norwich has brought home to the populace of Norfolk the needs of the hospital in no unmistakable manner, and the hospital authorities consider that the reopening of the ward will catch the rebound of the enthusiasm shown for the hospital when the ward was first closed.

In our issue of February 26 we published a short article on the plan adopted at Norwich for enlisting the support of the district by organising entertainments, etc. We understand that it is due to the success of this plan and the growth of the Sunday and Saturday Funds for obtaining regular contributions of 1d. per week that the hospital's finances have so greatly improved. From entertainments the hospital has received sufficient to make up the deficiency of £250 per week, £6,000 being received from this source in six months, and it is anticipated that the contributory scheme of the Sunday and Saturday Funds will bring in over £10,000 to the hospital this year. We hope to publish the details of this contributory scheme when the result of the six months' working is known. Meanwhile, we congratulate the authorities on their handling of the situation and on the results so far achieved, which should encourage all who have taken part to go forward with renewed vigour and confidence.

